# Does financing for private maternity services improve birth experiences in Poland? A mixed-methods study of the Babies Born Better Survey

**DOI:** 10.18332/ejm/195381

**Published:** 2024-11-12

**Authors:** Hanna Kacprzyk, Maria Węgrzynowska, Barbara Baranowska, Piotr Połomski, Marie-Clare Balaam

**Affiliations:** 1 Department of Gynecology and Obstetrics Didactics, Medical University of Warsaw, Warsaw, Poland; 2 Department of Midwifery, Centre of Postgraduate Medical Education, Warsaw, Poland; 3 Department of Psychometry and Statistics, Institute of Psychology, University of Gdansk, Gdansk, Poland; 4 School of Nursing and Midwifery, University of Central Lancashire, Preston, United Kingdom

**Keywords:** Poland, quality of care, birth experience, private service, babies born better survey

## Abstract

**INTRODUCTION:**

Women in Poland, despite having access to publicly-funded medical care during pregnancy, childbirth and the postpartum period, frequently use private care. Women's experience and satisfaction with childbirth have been considered one of the key indicators of the quality of care. In this study we explore whether and how paying for private childbirth services affects women’s experiences and satisfaction with care. The qualitative portion seeks to understand how individual women construct meaning around their childbirth experiences, including their relationships with healthcare personnel, medical interventions, birth environment, and professionalism.

**METHODS:**

This mixed-methods study is based on data from 951 online questionnaires completed by women who gave birth between June 2017 and June 2022, in Poland. This study is part of the international Babies Born Better Survey project. The project used simultaneous quantitative and qualitative data collection, it was exploratory with equivalent status of qualitative and quantitative data. Quantitative data were analyzed descriptively and chi-squared tests were conducted to compare women who used private and public care. Qualitative data were analyzed using inductive thematic analysis. The quantitative and qualitative results were integrated, in accordance with the chosen mixed-methods design.

**RESULTS:**

There were no major differences in sociodemographic characteristics (except living standards), health status and satisfaction with labor between women who paid for private services during childbirth and those who used only publicly-funded care. For both groups of women, healthcare personnel and their behavior were the most frequently mentioned aspect shaping childbirth experiences. Other important aspects were: medical interventions, birth environment, and staff professionalism.

**CONCLUSIONS:**

Although accessing private perinatal services care did not provide women with care consistent with their expectations, women put a lot of trust into private services as a means to receive more attentive care. Further research investigating the interplay between private and public services is needed to explore the question how private services may impact the care women receive and why women put so much trust in these services.

## INTRODUCTION

Women’s satisfaction and experiences of labor and care have recently grown in importance as a criterion for evaluating the quality of care^1,2^. Understanding the perspectives of women and the aspects of care that affected their experience, can determine the direction of changes to be made to improve the quality of perinatal services.

Previous studies conducted in different parts of the world have shown that the form of financing care during pregnancy, labor, and postpartum have a substantial impact on its quality and on women’s experiences of and satisfaction with childbirth. Women who used private perinatal care were more likely to undergo medical interventions such as episiotomy, elective cesarean birth, instrumental birth, labor induction and epidural^3-9^. For example, in a study from Brazil cesarean section accounted for 86.1% of all labors in private obstetric care and for 29.9% in public care^10^. Despite higher rates of medical interventions, researchers have also suggested that women who used private maternity services experienced higher level of autonomy and respect from healthcare personnel^11^.

Women in Poland have the right to publicly funded healthcare during pregnancy, childbirth and postpartum^12^. This care includes doctor-led prenatal consultations, hospital care (including childbirth) and postnatal midwife visits. Many of the prenatal care clinics in Poland are located in the community and not associated with any particular hospital. This means that the choice of the prenatal care provider does not determine the choice of the hospital for the birth. As a result, while many women attend private prenatal care, the overwhelming majority of births take place in publicly funded hospitals^6^. Private hospitals are few in number and very costly. However, some publicly funded hospitals offer additional private services on the top of the state-funded standard care. These include dedicated midwifery or doctor intrapartum care, high-standard postpartum recovery rooms, the presence of a companion during labor, private labor room, water birth, and, until recently, epidural during vaginal birth^13^. Over the last few years, as a result of public campaigns, both epidural during vaginal birth and the presence of a companion in most hospitals became a standard care not requiring any additional fees.

In 2010, the report outlining the functioning of maternity units in Poland, the Supreme Audit Office (SAO, Polish: Najwyższa Izba Kontroli, NIK) criticized the charging of these fees, except for dedicated midwifery care, as unjustified and violating the right to equal access to healthcare^14^. However, some of these services remain available on a fee-for-service basis in some publicly funded hospitals. According to the 2018 report of the Childbirth with Dignity Foundation, a non-governmental organization that monitors perinatal care in Poland, one in ten women participating in the survey incurred additional costs related to childbirth, and 3.1% paid for private midwifery services^13^.

Evidence from Baranowska et al.^15^ highlighted that comprehensive information, support and respect offered by the healthcare personnel as well as the birth environment had most impact on women’s satisfaction and their perinatal experience. All these aspects need improvement in Poland. As shown by the recent Childbirth with Dignity Foundation report, 24% of women who participated in the nationwide study experienced inappropriate comments from the healthcare personnel, 20% women felt being patronized, and 12% were screamed at during their hospital stay^13^. Even though most women in Poland birth in single labor rooms, more than 50% of hospitals reported still having shared labor rooms with no or minimal support equipment such as birthing balls^13,16^. This mismatch between women’s needs and the quality of available services is an important factor leading women to access private services in Poland.

Only few studies addressed the role of private services in the Polish maternity care. Previous research reveals that women pay for private care mainly to build personal connections with healthcare personnel and through these connections access more attentive care^6,17^. However, little is known about what women perceived as more attentive care and whether this differs between women who access publicly funded care and those who pay for private services. Thus, in this study we aim to fill this gap by exploring the elements of care that women perceive as positive and those that need improvement, and seeking to answer the question of whether and to what extent these elements were influenced by the form of financing of perinatal care.

## METHODS

### Study design and setting

In our study we used data collected as part of the Babies Born Better Survey project (https://www.babiesbornbetter.org/)(BBB)– a long-term international collaborative study coordinated by the University of Central Lancashire and a group of coordinators from different countries. The survey was developed within the frame of two EU Horizon COST Actions (IS0907 and IS1405). The aim of the project is to examine the views and birth experiences of women around the world to improve maternal and childbirth care by finding out what works, for whom and in what circumstances.

The data used in this study were from the third wave of the survey which ran between 2020 and 2022, was translated into 25 languages and collected the experiences of women who had given birth in the preceding five years. The questionnaire comprised two parts: 1) closed-ended questions about sociodemographic characteristic and the birth; and 2) open-ended questions exploring women’s experiences. In total, the questionnaire contained 28 questions.

**Figure 1:**
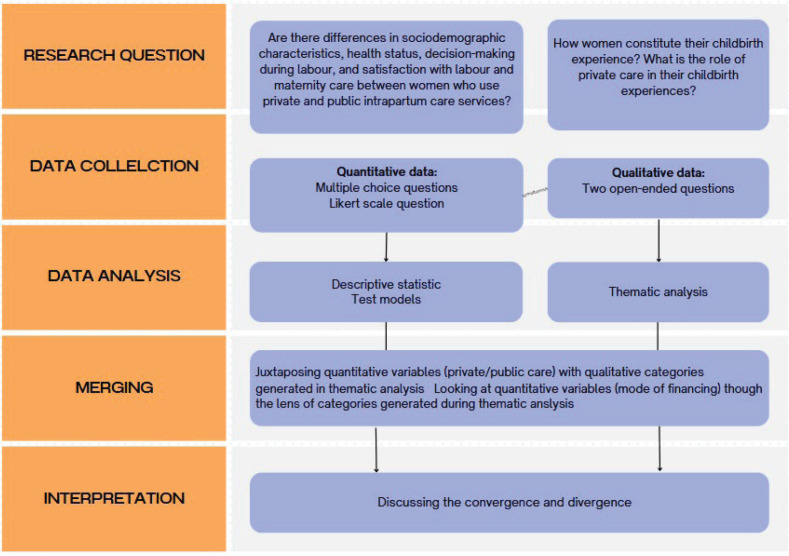
Diagram showing the type methodology of the study

In our study, we used a mix-methods approach in accordance with Figure 1. This approach, on the one hand, allowed us to see whether there is a correlation between the mode of financing of perinatal care and the health status (birth outcomes) and level of satisfaction (quantitative data analysis). On the other, it allowed us to explore what elements of care impact on women’s satisfaction (qualitative data analysis). By merging these two approaches, we aimed to deepen the knowledge on the extent that private and public services influence women’s perception of good care.

### Participants and recruitment

The questionnaire was disseminated through the project website (www.babiesbornbetter.org) and social media. The sample was derived from unpaid online advertisement and snowball sampling. This method reflects a compromise between study aim and feasibility; alternative sampling methods were not free of the same or other limitations. Recruitment for the study took place between June 2020 and June 2022 in online forums. In our study we included a total of 951 questionnaires completed by women who gave birth in Poland within the last three years. The exclusion criterion was lack of consent for the study. In all, 931 questionnaires were completed in Polish, 17 in English, and 3 in the following languages: Norwegian, Lithuanian, and Russian. Questionnaires completed in the foreign languages were translated into Polish for the purpose of the analysis. Due to the subject of the study, we excluded questionnaires which did not specify how the birth was financed (n=14). We also did not include questionnaires filled in by women who had home births (n=35). We divided the responses into two groups based on the funding sources for the birth: public and private. The group of women whose care was publicly funded included women who declared that they had not incurred any additional costs related to childbirth. The group of women whose care was privately funded included women who declared that they had either self-funded the birth or its cost had been covered by private insurance, as well as those women who had paid for additional private services (such as dedicated midwifery care or high-standard recovery room) in publicly-funded hospitals. This group was therefore not restricted only to the women who gave birth inprivate hospitals. Responses from women who defined the financing method as ‘other’ and provided a description were analyzed, and, where possible, assigned to the appropriate group.

### Data analysis

*Quantitative data analysis*

Closed-ended questions were statistically analyzed. In both groups we analyzed the sociodemographic and birthing characteristics of the participants using the Mann-Whitney U test and the chi-squared independence test to establish the relationship between variables and compare percentage distributions. The Mann-Whitney U test was applied due to the statistically significant difference of the analyzed variables from the normal distribution (verified with the Kolmogorov-Smirnov test) and significant disproportions in the sizes of the compared groups of women). Exploratory and descriptive analyses (frequencies and percentages) were applied to the variables: sociodemographic profile, parity, and pregnancy-related problems, the way care is funded, the type of birth, birth during pandemics and the satisfaction of birth. Median, mean and standard deviation were applied to age. For all statistical analysis we determined significance at the level of 0.05. Statistical analysis was performed using the Statistical Package for Social Sciences (SPSS®) version 24.

*Qualitative data analysis*

Next, we analyzed the responses to the following open-ended questions: ‘Imagine you are talking to a very close friend or family member who is pregnant, and that she is trying to decide where to give birth to her baby. She asks you what you think about the place you gave birth. Please answer her by finishing one or both of the following sentences: ‘I think you should give birth at the place where I did because...’,‘I think you should not give birth at the place where I did because…’. There were 3 data coders involved (HK, BB, MW). The subjects were obtained from the data. Data were coded manually without special software used for data management. We assigned these responses to the three categories: the first included responses recommending a particular place of birth, the second included responses which did not recommend it, and the third included questionnaires with arguments for and against a particular facility. Women rated their labor satisfaction on a five-point scale, with 1 indicating mostly very bad experience and 5 indicating mostly a very good experience very good experience. We statistically analyzed the number of responses in the three categories using absolute and relative frequencies (%), and we applied the chi-squared test of association and comparison of percentage distributions.

We applied thematic analysis to the remaining open-ended questions: ‘In the place where you gave birth, what were the three most positive experiences of your care?’, and ‘What do you think could have made your experience better?’. Up to three responses could be given to each question. The descriptive responses were read twice to identify and code the main themes. The first author identified potential themes and then grouped them into 10 broad categories (care, rapport, atmosphere, companion, safety, professionalism, procedures, birth environment, subjective approach, contact with the child). Then, we reviewed the potential themes and categories and discussed them with the other authors, and some were re-grouped. Finally, we created 4 main categories: ‘care, attention and rapport’, ‘medical procedures and forms of care’, ‘birth environment’, and ‘professionalism’.

*Mixed-methods analysis*

**Figure 2:**
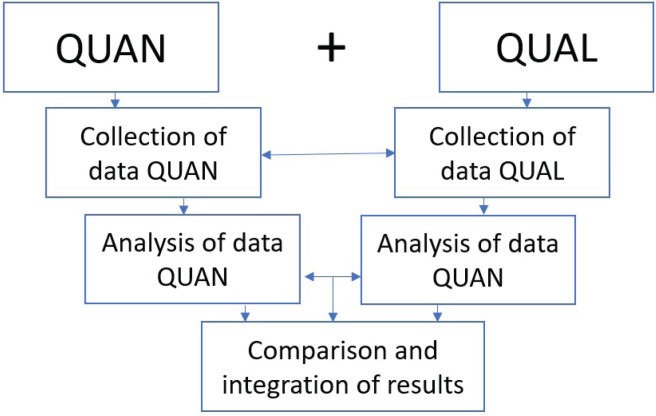
Mixed-methods design

The collection of quantitative and qualitative data took place in parallel and was exploratory. It was a project with equivalent status of qualitative and quantitative data. Integration of the two methods occurred at the data analysis stage (Figure 2).

**Figure 3:**
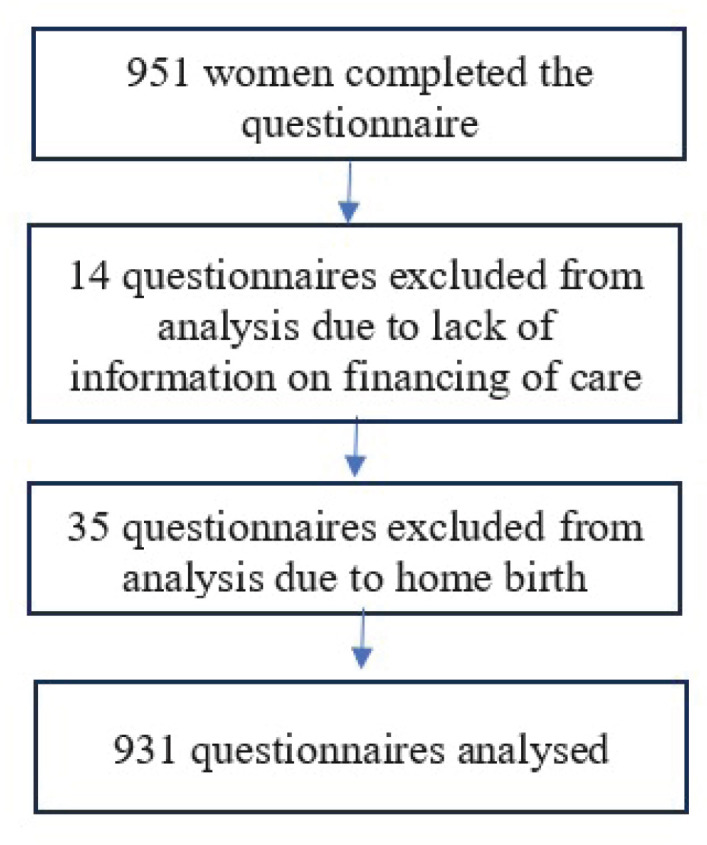
Collection of data

*Rigor*

The confirmability of our investigation is substantiated by the validity of our analysis, which was augmented through our reflexive documentation, by engaging in discussions regarding codes and themes among the authors, and by employing the participants’ expressions to exemplify our themes. Moreover, through transferability, we achieved via a meticulous delineation of the context surrounding our survey and the demographic characteristics of the respondents; and dependability through a comprehensive account of the procedures we executed in conducting the study.

*Ethical considerations*

Prior to accessing the questionnaire, participants were provided with information about data protection and privacy, and were informed that by completing the survey they were consenting to participate. Researchers had no previous contact with the participants. The researchers are experienced in conducting qualitative research. Ethical approval for the study was obtained from the ethics committee at the University of Central Lancashire (Unique reference: STEMH 449). No additional ethical clearance was required. Ethics approval was received on the June 2020, by the University of Central Lancashire Ethics Committee.

## RESULTS

### Quantitative results

The sample comprised 951 women in total, all of whom were aged about 30 years (median=30; mean=29.93; SD=4.36), women aged 29 years predominated (n=94, approximately 10%). Out of these 951 women, 86% (n=818) used publicly funded intrapartum care, while 14% (n=133) paid for some form of private care during labor and their subsequent hospital stay.

*Sociodemographic characteristics*

The groups were statistically significantly different in terms of socio-economic status. Women who used private care were significantly more likely to rate their living standard as better or much better than average (U=48301; Z= -2.230; p<0.05). There were no statistically significant differences between the groups in terms of employment status. Most of the women were employed or self-employed (82.2%), 10.7% were unemployed, a small proportion (1.4%) were students, and 5.7% reported their status as ‘other’. There were also no differences between the groups in terms of education level. Most women had tertiary education (86.2%), 10.5% had secondary, and 2.8% had vocational education. Only a few women declared that they had primary education (0.4%). The age differences between the responders were statistically significant (U=47653.5; Z=-2.301; p<0.05), with women opting for privately funded care being older (mean age: 30.85 years) than those who received publicly funded care (mean age: 29.78 years); 58% of women were primiparous and 42% were multiparous. There was no statistically significant correlation between parity and the way care was funded.

*Health status*

There was no correlation between the way care was funded and the gestational age at birth, the occurrence and type of problems during pregnancy or the type of birth facility. This would suggest that women with a high-risk pregnancy were equally likely to seek private intrapartum care as women with a low-risk pregnancy. In all, 64.1% of women in our sample had a vaginal birth, 16.7% had an emergency cesarean section, 16.0% had an elective cesarean section, and 3.2% had an instrumental labor (ventouse or forceps). There were also no statistically significant differences in the mode of birth between the groups. As 56% of participants gave birth during the COVID-19 pandemic, we also examined differences in the type of financing of care before or during the pandemic, but these were not statistically significant.

*Decision making during labor*

In our survey, we asked women about decision-making during labor and birth. Excluding cesarean section responses (where doctors make decisions), 73.7% of women reported midwives as primary decision-makers (63.7% in public funding, 10% in private care). Shared decision-making between midwives and doctors was indicated by 15.1% (12.8% public, 2.3% private), while 7.2% pointed to doctors (5.1% public, 2.2% private). Additionally, 4% responded ‘other’ (2.3% public, 1.7% private). Although the survey did not include ‘I’ as an option, 3.2% (n=21) elaborated in open-ended responses, asserting themselves as primary decision-makers (1.7% public, 1.5% private). Women using private care emphasized midwives’ role in decision-making, considering the disparity in funding group sizes.

*Satisfaction with labor and maternity care*

There was no statistically significant association between satisfaction and care funding. Private care had an average score of 3.94, while public care scored 3.83. When asked about recommending their birth facility, 66.3% of women recommended it, 11.7% advised against it, and 22.1% provided arguments for and against. Although private care recipients were slightly less likely to advise against a specific facility (1.7% vs 9.9% for publicly funded care), funding did not significantly impact childbirth experience quality.

### Qualitative results

We thematically analyzed the two open-ended questions included in the questionnaire: ‘In the place where you gave birth, what were the three most positive experiences of your care?’ and ‘What do you think could have made your experience better?’. In total, we collected 3479 answers (respondents could provide up to three answers per question). Of these, 2187 answers referred to the question about the most positive aspects of care, and 1283 concerned elements of care that women would like to change.

We identified four categories: ‘care, attention and rapport’,‘medical interventions and forms of care’, ‘birth environment’, and ‘professionalism’. The number of responses for each category were similar in both groups of respondents (Table 1).

**Table 1:** Responses to questions grouped by thematic category and the mode of funding

*Thematic category*	*Privately funded care*				*Publicly funded care*					
	*Most positive aspects of care n*	*What could be improved n*	*Total n*	*Response rate in each category %*	*Most positive aspects of care n*	*What could be improved n*	*Total n*	*Response rate in each category %*	*χ^2^*	*p*
Care, attention and rapport	187	61	248	50	1069	407	1476	49.5	0.952	0.337
Medical interventions and forms of care	50	65	115	23.2	333	332	665	22.3	1.707	0.202
Birth environment	46	48	94	19	283	246	529	17.7	0.666	0.421
Professionalism	24	12	36	7.3	184	88	272	9.1	0.014	0.907
Other	2	1	3	0.6	18	23	41	1.4	0.584	0.421
Total	309	187	496	100	1887	1096	2983	100		

*Care, attention and rapport*

Among the responses, 49.6% highlighted positive aspects (36.1%) and areas for improvement (13.5%) related to childbirth experiences. Women emphasized care, attention, and rapport. They evaluated the competences of the entire birth team, including midwives, obstetricians, and other professionals. Key themes included support, respect, empathy, and feeling safe. Those aspects most significantly influenced women’s experiences. Midwives received the most frequent praise, while other team members were mentioned less often:

*‘[The most positive experience was] in the first stage of labor, when midwife praised me and supported me by saying I would make it’.*

Lack of attention and empathy was also most frequently listed as an aspect that needed improvement. Women complained about the lack of respect for their intimacy, decisions and needs. Women also highlighted that the staff were impolite when addressing them:

*‘[It would have been better] if the staff had taken my opinion into account and respected it, without shifting to a negative attitude if something was not to their liking’.*

No help with childcare and insufficient availability of healthcare professionals were frequently identified as aspects of care that needed improvement:

*‘[My experience could have been improved by] support from midwives and doctors working in the ward after my baby was born’.*

Women emphasized the positive impact of having a chosen birth companion, allowing fathers to provide kangaroo care for the baby after a cesarean section, and enabling visitors to assist with infant care and maternal self-care. However, during the COVID-19 pandemic, restrictions on companions and visitor bans were seen as negative factors.

Women often viewed private maternity care during labor as a guarantee of success and quality. References to private care as a key to positive birth experiences appeared both in positive aspects (expressing satisfaction with paying for private midwifery care) and in aspects that needed improvement (speculating that their experience could have been better with a private midwife):

*‘If I hadn’t had private midwifery care, I would have feared it wouldn’t have gone so smoothly. While waiting for a laborroom only the midwife was interested in my condition’.*

*‘[My experience could be improved by the] care provided by a paid midwife’.*

*Medical interventions and forms of care*

Women frequently described medical interventions and their impact on childbirth experiences. Negative assessments of medicalization ranged from minimally invasive procedures (like routine iv insertion) to major interventions (such as the Kristeller maneuver). Access to or lack of access for certain procedures was also mentioned. Positive aspects included perineal protection, skin-to-skin contact, mobility during labor, and lactation care. Unrestricted movement, vertical positions, water immersion, and access to equipment like birthing balls were praised. Long cardiotocographic monitoring (CTG) and limited mobility was seen as needing improvement. Oxytocin use during labor was viewed with mixed feelings, with most negative feedback related to restricted movement it usually entailed. Women expected less invasive induction methods and expressed dissatisfaction with directed pushing:

*‘Freedom of movement during the CTG instead of lying down. This should be the norm. Meanwhile, having to lie down is the norm, and if you want to get out of bed, you have to fight for it. This is especially true in the second stage of labor, when women are advised to remain in a semi-reclining or supine position for safety reasons’.*

Lactation care significantly influences birth experiences. Women appreciate immediate breastfeeding assistance, certified lactation counselors, and responsive midwives. The absence of these elements was seen negatively. Some women mentioned formula feeding without consent and lack of lingual frenulum evaluation as areas that needed improvement:

*‘[My experience could have been improved by] midwives not forcibly putting the baby to the breast when the nipples are already covered with sores’.*

The elements of care that women most often appreciated were: vertical position and freedom of movement, lactation care, skin-to-skin contact after labor, alternative pain relief methods, and respectful consideration of their birth plan. Absence of these elements of care, lengthy procedures during admission and failure to respect the birth plan were criticized.

*Birth environment*

Another aspect that had a strong influence on shaping birth experiences was the birth environment, including the conditions in the rooms and the availability of beds and medical staff. Positive and negative experiences connected with this aspect of care were reported with similar frequency (9.5% and 8.5% of total responses, respectively). Women most frequently mentioned supporting equipment such as birthing balls in the rooms, décor and furnishings, ensuite bathrooms, cleanliness, and adaptability of the amenities to patients’ needs. An element that had a negative impact on women’s satisfaction were shared labor rooms. Women also expressed dissatisfaction with the lack of personal hygiene products:

*‘The conditions in the postnatal ward were terrible, it was cold, dark, bathrooms not renovated for probably 30 years, no bathroom in the room’.*

Women’s birth experiences were also influenced by organizational issues such as shortage of staff, hospital beds, and queues in the admission room. Women mentioned feeling anxious about being sent to another hospital, as they believed shortages of beds were common:

*‘Fewer number of patients should be admitted for labor, the hospital could not cope with the number of patients in laborrooms, and I had to stay in the pathology ward for 7 hours because the laborrooms were full’.*

Women were concerned with the quality of food in hospitals, including the size and freshness of meals, as well as the lack of catering for individual dietary needs. The high number of responses commenting on food implies that this was a factor that has a strong impact on the level of satisfaction:

*‘…food! - a woman who gave birth late in the evening or at night has to wait until 8 or 9 a.m. for breakfast. Quality of meals very poor’.*

Interestingly, both groups of women (private and public funding) negatively perceived having to pay for private services to improve their care. Dedicated midwifery care and good conditions were seen as services every woman deserves at no extra cost. Some women claimed that the possibility of giving birth at home or in a midwife-led birth unit could have improved their satisfaction. At the same time, they pointed out there is no reimbursement for home births:

*‘[My experience could have been improved by] not having to pay to get decent conditions’.*

*Professionalism*

In this category, a large share of the positive responses comprised general statements about the professionalism, experience, expertise, and efficiency of medical staff. Women’s satisfaction was positively influenced by the experience of feeling safe and by effective interventions performed by medical staff:

*‘Quick decision to perform a cesarean section, saving the baby’s health and life’.*

Women’s satisfaction was also shaped by the respect for their rights, especially the right to informed consent for medical procedures. Recognizing women’s preferences and taking them into account were important factors which could have enhanced their birth experience. Women attached great importance to being informed about the medical procedures that were undertaken:

*‘If only I had had an opportunity to talk to a doctor about possible solutions and their consequences. What I lacked was reliable information’.*

## DISCUSSION

Our research shows that there were no major differences in sociodemographic characteristics (except living standards), health status, birth outcomes and satisfaction with labor between women who paid for private services during childbirth and those who used only publicly funded care. Integration of quantitative and qualitative data at the level of analysis showed that for both groups of women, healthcare personnel and their behavior was the most frequently mentioned aspect shaping childbirth experiences. Apart from that, women also mentioned medical interventions, birth environment and staff professionalism as other important aspects, and there were no statistically significant differences in the frequency that these aspects were mentioned in both groups. Despite the last differences, thematic analysis of the qualitative data showed that women perceived private services as a guarantee of more attentive care. As far as we know, this is the first study in Poland that seeks to elicit the differences between the actual elements of birth experiences between women who pay for private care and those who use publicly funded care.

Regardless of how the birth was financed, women believed that the aspects of care that required improvement the most, were staff attitudes and the quality of rapport between the staff and women, particularly communication. Women valued the politeness of the staff and their ability to pass on information, but, at the same time, they wanted to be given recognition, meaning the staff should recognize and respect their decisions. Researchers obtained similar results in the BBB surveys in other countries. In the survey conducted in Croatia, staff kindness was the second most frequently mentioned category which influenced women’s positive experience of childbirth, afterbirth environment^18^. It should be noted that in that study qualitative responses were divided into 18 categories, therefore many aspects of interpersonal care and interactions between woman and staff, such as understanding, emotional and informational support, and respecting a woman’s wishes, were separate categories^18^.

The BBB survey conducted by researchers in Lithuania found that in order to improve the elements most distressing for women, it was not innovative technical solutions that were needed, but rather a strengthening of the emotional and interpersonal aspects of care^19^. The authors of the study from Cyprus also showed that medical personnel’s commitment in the process of birth and respect for the woman’s need, had a positive impact on woman’ sexperience^20^. Those studies also showed strong association between staff behavior and women’s negative birth experiences, stressing the need to pay particular attention to these aspects when planning and shaping care for women in labor^18,20^.

Despite several studies indicating that good rapport with medical personnel, their support and mutual trust are of key importance for women’s experience of childbirth^21,22^, the situation of women giving birth in Poland is far from ideal. As mentioned earlier, verbal and other forms of abuse remain a widespread problem in maternity hospitals in Poland^13^. To minimize the risks of mistreatment, women with financial resources pay for private care in Poland, particularly private dedicated midwifery care^17^. This allows women to get to know the midwife, discuss their needs before the birth, and find out what the midwife can offer. Paying for midwifery care during labor, or paying for private prenatal visits, is an attempt to secure rapport with a staff member and assure continuity of care. As researchers showed, establishing rapport before birth, and especially ensuring continuity of midwifery care, has a positive effect on the quality of provided services and the level of satisfaction of the woman^23,24^.

However, as a previous study showed^6^, and our study partially confirms, private care, while bringing some benefits, does not protect women from mistreatment and does not increase the level of satisfaction with postnatal care. The quantitative results of our study also showed no significant differences in women’s satisfaction and experience of childbirth between the group that paid for at least some elements of maternity care and the group that received only publicly funded care. Women in both groups equally often cited the issues related to care, attention and rapport as areas that required improvement. At the same time, our thematic analysis of the descriptive responses suggests that women perceived privately funded care as a promise to quality services and good birth experience. In the ranking of hospitals carried out in 2022 by the Childbirth with Dignity Foundation, two of the top ten hospitals with best scores from women, were private facilities. Given the small number of such facilities in Poland, it shows that Polish women have a high level of trust in private hospitals^25^.

The discrepancy between levels of satisfaction and women’s perception of what private care offers may be due to several reasons. Firstly, as various forms of financing overlap in Poland, it is quite difficult to pinpoint who is a private patient. Women who receive partially private intrapartum care remain in the same system, facility and often room as women whose care is fully publicly funded. Second, as mentioned previously, many women attend private prenatal consultations to obtain the status of a private patient that then transfers through personal relationships with a care provider to publicly funded wards. These women may hold a status of a ‘private patient’ and receive better care although they do not pay for private services in the hospitals and, in our study, would be categorized as ‘publicly funded care’.

This demonstrates that while private services bring some benefits to women who can access them, the bulk of care, even for women who pay for private services, is provided within the fragmented, underfunded and understaffed publicly funded care. In addition, the availability of private services themselves can contribute to the further fragmentation and understaffing of publiclyfunded care. This study provides a very valuable insight into what aspects of care shape women’s childbirth experiences. Further research investigating the interplay between private and public services and the variety of their forms is needed. In particular, looking into ways women obtain their ‘private patient’ status or what exactly is considered ‘private care’ may provide a very valuable perspective into what influences quality of care that women receive.

### Strengths and limitations

A strength of our study is the number of participants. An online survey allowed nation-wide dissemination obtaining responses from women across the country, resulting in data that included a wide variety of experiences. The use of an online survey may have attracted respondents with higher internet literacy and engagement, potentially leading to a sample skewed toward more educated or technologically proficient women, which could affect the generalizability of the findings (selection bias). Furthermore, since the study relies on participants’ self-reported birth experiences, there is a possibility of recall bias. Another limitation is the small proportion of private-care users in the sample. By conducting both quantitative and qualitative analysis, and analysis of closed-ended and open-ended questions, we were able to obtain comprehensive data from a large number of respondents. A major advantage of the BBB survey is its international nature, which makes it possible to compare data collected in different countries.

## CONCLUSIONS

There were no significant differences in the health status, clinical outcomes and levels of satisfaction between women who paid for private services and those who used standard care. Both groups of women similarly frequently mentioned the behavior of healthcare personnel as the most important factor influencing (negatively and positively) their levels of satisfaction. At the same time, women considered private services as a guarantee of more attentive care. This discrepancy is consistent with our previous studies showing that women’s hopes and use of private services do not influence women’s levels of satisfaction with care^6^. Further research investigating this interplay between private and public services is needed.

## Data Availability

The data supporting this research are available from the authors on reasonable request.
